# Penalized logistic regression with low prevalence exposures beyond high dimensional settings

**DOI:** 10.1371/journal.pone.0217057

**Published:** 2019-05-20

**Authors:** Sam Doerken, Marta Avalos, Emmanuel Lagarde, Martin Schumacher

**Affiliations:** 1 Institute of Medical Biometry and Statistics, Faculty of Medicine and Medical Center, University of Freiburg, Freiburg, Germany; 2 Freiburg Center for Data Analysis and Modeling, University of Freiburg, Freiburg, Germany; 3 University of Bordeaux, Inserm, Bordeaux Population Health Research Center, UMR1219, Bordeaux, France; 4 SISTM team, INRIA Bordeaux-Sud-Ouest, Talence, France; Universitat de Valencia, SPAIN

## Abstract

Estimating and selecting risk factors with extremely low prevalences of exposure for a binary outcome is a challenge because classical standard techniques, markedly logistic regression, often fail to provide meaningful results in such settings. While penalized regression methods are widely used in high-dimensional settings, we were able to show their usefulness in low-dimensional settings as well. Specifically, we demonstrate that Firth correction, ridge, the lasso and boosting all improve the estimation for low-prevalence risk factors. While the methods themselves are well-established, comparison studies are needed to assess their potential benefits in this context. This is done here using the dataset of a large unmatched case-control study from France (2005-2008) about the relationship between prescription medicines and road traffic accidents and an accompanying simulation study. Results show that the estimation of risk factors with prevalences below 0.1% can be drastically improved by using Firth correction and boosting in particular, especially for ultra-low prevalences. When a moderate number of low prevalence exposures is available, we recommend the use of penalized techniques.

## Introduction

The modeling of binary outcomes plays an important role in epidemiology. It is often applied when studying the influence of risk factors on a health outcome of interest, e.g. death or the onset of a disease. Logistic regression is the standard method for evaluating such data. It is a popular method of choice because it allows quantification of risks in terms of odds ratios that are easy to interpret. Further, it provides the basis of many variable selection strategies to determine which suspected risk factors are relevant and which are not [1–3].

One challenge in using logistic regression, however, is the analysis of binary risk factors with very low prevalences, e.g. a rare exposure that is either present or absent in individuals. When estimating risk factors, low prevalences can lead to numerical problems because the likelihood function of the logistic regression model may not converge. This can result in biased coefficient estimates, or estimation may not be possible at all [4, 5]. All things being equal, a lower prevalence of exposure leads to an increase in bias, higher variability, wider confidence intervals and a loss of power. In short, lower prevalences lead to a loss in accuracy and precision of risk estimates.

In epidemiology, rare risk factors with low prevalences are a common and recurring problem. [6] present a case-control study on cervical cancer, in which only about 1.9% of the patients were positive for the risk factor HP virus types 6/11. In another example, [7] describe a case-control study on risk factors for childhood cancer, with a prevalence of 0.4% of mothers who smoke more than 20 cigarettes per day. Even though the associated odds ratio of 4.5 has an extremely large 95% confidence interval (0.5-39.0), the plausibility of the estimates is not discussed. Yet, despite its ubiquity and relevance, the problem of rare risk factors is often ignored or not recognized [8]. [9] gave an overview and assessment that rare exposures substantially overestimate the risk of adverse drug reactions. Observational studies on environmental or occupational exposures may be particularly prone to low prevalences.

Methods to deal with rare risk factors tend to be ad hoc. A common technique to circumvent rare risk factors is to combine the rare exposure with other related exposures, but this prohibits any inference about the rare exposure. In other settings, when rare exposures are encountered in a simple setting of a contingency table, continuity corrections that go back to [10] are common, but generalizations that allow for multivariable analysis are less known. Resampling methods may be used to oversample observations with the rare exposure, but this presumes knowledge of the true underlying distribution of risk factors in the target population, which in practice is often unavailable. Propensity score methods may also be used to balance infrequent exposures, but are presumed to not be effective for very low prevalences, i.e. below 1% [11]. Since the focus of this paper is multivariable risk factor analysis, no knowledge of the true underlying distribution of risk factors is presumed and there is more than one single target risk estimate on which to balance confounders. Therefore, these methods are not considered here.

A modern analysis tool enjoying increased popularity in recent years is penalized regression. This method constitutes an improvement upon the maximum likelihood method under certain circumstances. If *ℓ*(***β***) is the log-likelihood function of a regression coefficient vector ***β*** for the risk factors under investigation, then the idea of penalized regression is to modify the log-likelihood by adding a penalization term Π(***β***) to estimate the coefficients: ℓ*(***β***) = *ℓ*(***β***) + Π(***β***) [12].

Penalized regression has become very popular for analyzing so-called high-dimensional datasets where the number of variables *p* far exceed the number of observations *n*, making estimations using classical statistical methods similarly challenging. While penalization methods have become common practice for the analysis of high-dimensional data, we show that the methods are also useful for low-dimensional settings.

The application of penalized regression in the medical or epidemiologic literature still seems limited to single instances [13]. However, there is increased awareness that new statistical techniques need not only be developed but also assessed more thoroughly to guide their usage. This has culminated in an initiative that explicitly encourages comparison studies of methods [14].

In a previous work, [15] studied penalized regression for estimating low-prevalence risk factors in the context of the case-crossover design. In this paper we do this in the context of an unmatched case-control study which makes three important contributions. First, from an epidemiological perspective, case-crossover studies have a different area of application compared to case-control studies and thus are relevant to different fields of research. A characteristic difference is that case-crossover studies control for time-invariant confounders by design, while case-control studies may be able to control for time-varying confounders. Secondly, from a statistical perspective, the standard analysis tool for case-crossover is the conditional logistic regression model, whereas case-control studies without matching usually use unconditional logistic regression which uses a different likelihood function. The effective sample size is determined by the number of cases with discordant exposures in case-crossover studies, and by the number of cases versus controls in the case-control study. As we aim to provide recommendations for epidemiologists, the differences between the two designs matters and justifies separated studies. And lastly, [15] make comparisons to a case-control study as the gold standard which leaves uncertainty about the true estimates; in this paper, we included a simulation study where the true estimates can be known.

We use data from a study on road traffic accidents in France between 2005 and 2008, where the risk factors were prescription medications. 117 of the 234 drugs under investigation had a prevalence of less than 0.1% in the cohort, and standard methods fail to produce plausible risk estimates for these drugs. We modeled a simulation study after the case-control study to gain better insight into the strengths and weaknesses of penalization methods. The qualities of penalized regression were studied to assess their performance for risk estimation and variable selection in epidemiology in the presence of low-prevalence risk factors.

## Materials and methods

### The logistic model and different estimation methods

We study logistic regression, a classical technique for risk factor analysis, and compare penalized techniques aimed at improving estimation when risk factors have low prevalences. The goal is to fit a model for a binary outcome *y* in terms of *p* risk factors ***x*** = (*x*_1_, …, *x*_*p*_), i.e. to fit a model for *π*(***x***) ≔ *P*(*y*|*x*_1_, …, *x*_*p*_).

The model considered here is multivariable, which will not be specifically mentioned anymore further on. The terms ‘risk factors’ and ‘variables’ are used interchangeably.

#### Logistic regression

We consider a logistic regression model

$$\begin{array}{c} \pi \left(x\right)={\mathrm{logit}}^{-1}(\beta_{0}+\sum_{j=1}^{p}\beta_{j}x_{i}) \end{array}$$

where logit^−1^ is the inverse of the logit transformation, with regression coefficients ***β*** = (*β*_0_, …, *β*_*p*_). The regression coefficients are determined by maximizing the log-likelihood function *ℓ*(***β***) over *n* observations,

$$\begin{array}{c} \ell \left(\beta \right)=\sum_{i=1}^{n}[y_{i}\log\left(\pi \left(x_{i}\right)\right)+\left(1-y_{i}\right)\log\left(1-\pi \left(x_{i}\right)\right)], \end{array}$$

using standard maximum likelihood methods. This yields a system of *p* + 1 equations which is solved using Newton-Raphson-like methods, for example the iterative weighted least squares procedure [12]. Its risk factor estimates are the regression coefficients. To select which variables are considered relevant [16], a simple procedure is backward elimination by successively removing variables with the highest p-value and re-fitting the model until all remaining variables have a p-value below 0.157. Using a p-value threshold of 0.157 yields similar results to variable selection based on the AIC and thus may be used as a substitute [17]. All variables left in the model after elimination are considered selected. Variables for which the likelihood function failed to converge are considered not selected.

#### Firth

A Firth correction introduces a penalty based on the observed Fisher information matrix *I*(***β***),

$$\begin{array}{c} \ell^{\text{Firth}}\left(\beta \right)=\ell \left(\beta \right)+\frac{1}{2}\log\left(\det I\left(\beta \right)\right), \end{array}$$

to estimate risk factors [4, 18]. The penalty term is maximized when *π*(***x***) = 0.5 which is maximized when ***β*** = **0**, thus the introduction of the penalty terms shrinks the coefficients towards 0. Like for the standard logistic regression model, we base variable selection on backward elimination with a p-value threshold of 0.157. P-values are calculated based on the profile penalized log-likelihood. All variables left after elimination are considered selected.

#### Lasso

The lasso estimation method uses a penalty term of the form

$$\begin{array}{c} \ell^{\text{lasso}}\left(\beta \right)=\ell \left(\beta \right)-\lambda \sum_{j=1}^{p}\left|\beta_{j}\right| \end{array}$$

[12, 19]. As the tuning parameter λ ≥ 0 increases, an increase in *β*_*j*_ becomes more “costly”, and as λ → + ∞, coefficients shrink towards 0. This has the advantage that risk factor estimation and selection are performed simultaneously, reducing the problem of overestimation. Using 10-fold crossvalidation once, the λ_min_ which minimizes the log-likelihood across all 10 partitions is chosen. All variables with nonzero coefficients for λ_min_ are considered selected.

#### Ridge regression

Ridge regression [12, 20] also shrinks the estimated coefficients towards zero:

$$\begin{array}{c} \ell^{\text{ridge}}\left(\beta \right)=\ell \left(\beta \right)-\lambda \sum_{j=1}^{p}\beta_{j}^{2}. \end{array}$$

The tuning parameter λ is again determined via 10-fold crossvalidation. Ridge regression was the first such penalized method which gained traction in statistical literature and it is closely linked with the lasso because of the similar penalization of the regression coefficients. Unlike the lasso, it does not have an inherent variable selection property. We therefore calculate 95% confidence intervals by taking 100 bootstrap samples from a dataset and calculate the ridge regression coefficients for each sample. If the interval of the 0.025 and 0.975 quantile of the regression coefficients includes the null effect 0, the variable is not considered selected, and vice versa.

#### Boosting

Component-wise likelihood-based boosting is an iterative, forward selection-type procedure where the likelihood function is approximated step by step [21]. It is very flexible and can be used to fit generalized linear models or generalized additive models [22]. Like the lasso, estimation and selection of risk factors is performed simultaneously as variables can receive a coefficient estimate of 0.

1. In a first step, a model with only an intercept term is fitted,
  $$\begin{array}{c} {\hat{\eta }}^{\left[0\right]}={\hat{\gamma }}_{0}. \end{array}$$
2. In each following step *m*, models ${\hat{\gamma }}_{j_{0}}+{\hat{\gamma }}_{j}x_{j}$ are fitted for every variable *j* = 1, …, *p*, using the fit of the previous model ${\hat{\eta }}^{\left[m-1\right]}$ as an offset:
  $$\begin{array}{c} \ell_{ML}^{\text{offset}}\left({\hat{\gamma }}_{j}\right)=\sum_{i=1}^{n}[y_{i}\log\left(\pi \left(x_{ij}|{\hat{\eta }}^{\left[m-1\right]}\right)\right)+\left(1-y_{i}\right)\log\left(1-\pi \left(x_{ij}|{\hat{\eta }}^{\left[m-1\right]}\right)\right)] \end{array}$$
  with $\pi \left(x_{ij}|{\hat{\eta }}^{\left[m-1\right]}\right)={\mathrm{logit}}^{-1}\left({\hat{\gamma }}_{0}+{\hat{\gamma }}_{j}x_{j}+{\hat{\eta }}^{\left[m-1\right]}\right).$ Using
  $$\begin{array}{c} j^{*}=\underset{j}{\arg\max}\;\ell_{ML}^{\text{offset}}\left({\hat{\gamma }}_{j}\right)-\lambda {\hat{\gamma }}_{j}^{2}, \end{array}$$
  the coefficient ${\hat{\gamma }}_{j^{*}}$ of the variable which increases the penalized likelihood
  $$\begin{array}{c} \ell_{\text{ML}}^{\text{offset}}\left({\hat{\gamma }}_{j}\right)-\lambda {\hat{\gamma }}_{j}^{2} \end{array}$$
  the most is updated to the model:
  $$\begin{array}{c} {\hat{\eta }}^{\left[m\right]}={\hat{\eta }}^{\left[m-1\right]}+{\hat{\gamma }}_{j_{0}^{*}}+{\hat{\gamma }}_{j^{*}}x_{j^{*}}. \end{array}$$
3. The regression coefficient estimate ${\hat{\beta }}_{j}$ after *M* boosting steps then equals the sum of the updated estimates:
  $$\begin{array}{c} {\hat{\beta }}_{j}=\sum_{m=1}^{M}{\hat{\gamma }}_{j}^{\left[m\right]}. \end{array}$$

The number of boosting steps *M* is the main tuning parameter. The penalty parameter λ will influence how many optimal boosting steps *M*_optimal_ are needed, but it needs to be chosen only very coarsely: [23] advise to use a λ-value which ensures that *M*_optimal_ ≥ 50. For modeling our data, a model is fitted using boosting with 500 boosting steps. With a measure for degrees of freedom [23], an AIC can be obtained, and the value of *M* that produces the lowest AIC is chosen as *M*_optimal_. All variables with nonzero coefficients are considered selected.

### Data

#### Case-control data

In order to compare unpenalized with penalized methods we apply them to a subset of data taken from the CESIR study [24]. CESIR was a registry-based unmatched case-control study conducted in France from 2005 to 2008. The sample consisted of drivers who were involved in a road traffic accident. The outcome of interest was whether the drivers were considered responsible for causing the accident as determined by a validated scoring technique. The risk factors under investigation were prescription medicines that were taken by the drivers at the time of the accident. Penalized regression has been studied before in the context of the CESIR study [25]. The distinction here is that we focus our analysis on the subpopulation of younger drivers between the age of 18 and 55 years for whom the prevalence of taking prescription drugs is particularly low compared to drivers older than 55 years. Thus the data consists of 50,728 drivers (26,586 cases and 24,142 controls) and 234 drugs without any missing values which potentially may influence the likelihood of causing an accident. Confounders such as age, sex, alcohol consumption, vehicle type, time of year, time of day, are also available and adjusted for in the analyses. All data is binary. Data taken from the CESIR study will be called case-control data.

#### Simulation data

A simulation study is created to emulate the CESIR study as closely as possible but on a smaller scale to ease computation. We consider 50 risk factor variables *x*_1_, …, *x*_50_ whose prevalences *P*(*x*_*i*_ = 1), or *P*(*x*_*i*_) in short, are Bernoulli distributed. Prevalences range from 0.005% (1 in 20,000) to 3% (1 in 33.3). The study was designed such that 10% of the risk factors are relevant, and the relevant risk factors are evenly spaced from most prevalent to least prevalent on a logarithmic scale. The relevant risk factors are thus *x*_1_ (most prevalent), *x*_13_, *x*_26_, *x*_39_ and *x*_50_ (least prevalent). To simulate low correlations, we assume a common probability for two risk factors *x*_*i*_, *x*_*j*_ of $P\left(x_{i},x_{j}\right)=P\left(x_{i}\right)P\left(x_{j}\right)2^{h_{i,j}}$, *h*_*i*,*j*_ normally distributed *h*_*i*,*j*_ ∼ *N*(0, 0.25), *i* = 2, …, 50, *j* < *i*. We also include confounders *z*_1_, *z*_2_ which represent unobserved factors that may influence driving ability, with prevalences of 50%.

The first step is to simulate a large population. Using the R package rmvbin [26], we simulate the risk factor and confounder exposures for *n* = 100, 000 observations with said prevalences and correlations of the risk factors *x*_1_, …, *x*_50_. For the outcome *y*, we also assume a Bernoulli distribution. The baseline probability of the outcome shall be 50%, thus the intercept term *β*_0_ = 0. The effect sizes *β*_*j*_ of relevant and irrelevant risk factors are ±1 and 0 respectively. Effect sizes *γ*_1_, *γ*_2_ of the unobserved confounders are chosen so as to produce a signal-to-noise ratio $\frac{Var(\sum \beta_{j}x_{j})}{Var(\sum \gamma_{j}z_{j})}=3$. They are of equal magnitude, i.e. *γ*_1_ = *γ*_2_, with one confounder being risk increasing while the other is protective of the outcome, therefore having opposite signs in the data-generating model. The outcome *y* is then simulated for each observation in the population based on the model

$$\begin{array}{cc} P(y|x_{1},...,x_{50},z_{1},z_{2}) & ={\mathrm{logit}}^{-1}\left(\beta_{0}+\sum_{j=1}^{50}\beta_{j}x_{j}+\gamma_{1}z_{1}-\gamma_{2}z_{2}\right)\\ & ={\mathrm{logit}}^{-1}\left(-x_{1}+x_{13}-x_{26}+x_{39}-x_{50}+\gamma_{1}z_{1}-\gamma_{2}z_{2}\right), \end{array}$$

where logit^−1^ is the inverse of the logit transformation. We then draw 5,000 cases and controls each from the population to comprise a simulation dataset. With a sample of *n* = 10, 000 and *p* = 50 risk factors, the sample size is smaller compared to the case-control data to lighten the computational burden, but the observation-per-variable ratio is kept the same.

This scenario is repeated to create 100 simulated datasets. These are called simulation data. A markdown file containing the R code used in creating the simulation data is available in S1 and S2 Files.

### Evaluation measures

In the case-control data, we fit a logistic regression model in the dataset of 50,728 observations using all risk factors and confounders as covariates. We call this the reference model and the resulting estimates of the risk factors are denoted as *β*_*j*_. Fitting the reference model, 21 risk factors had estimates with |*β*_*j*_| > 2. These estimates are beyond the boundaries of any confidence interval described by [24] and are therefore considered implausible. Although this might make logistic regression seem like an unsuitable reference method, we still chose to use it as our reference method because it is the standard technique for analyzing case-control data. Further, making comparisons to an unpenalized method clearly demonstrates the effects of penalization. The implausible estimates are used for fitting but will not be considered when making comparisons to the reference model, leaving 213 risk factors of interest. We continue by taking a random subsample approximately 1/10*th* the size with *n* = 5, 000 observations (with a 1:1 case-control ratio) and fit models using standard logistic regression and penalized regression to the subsample. Boosting was not included here due to excessive computational burden. The process is repeated with 100 random subsamples, and the estimates ${\hat{\beta }}_{j}^{b}$ from the *bth* subsample are averaged and denoted as ${\hat{\beta }}_{j}=\frac{1}{100}\sum_{b=1}^{100}{\hat{\beta }}_{j}^{b}$.

In the simulation data, the coefficients used for generating the data are the benchmark estimates and denoted by *β*_*j*_, and ${\hat{\beta }}_{j}$ denote mean estimated coefficients from the 100 simulation studies.

#### Bias and mean squared error

The bias of a risk factor is assessed through ${\hat{\beta }}_{j}^{b}-\beta_{j}$, and the mean bias is summarized using $\frac{1}{100}\sum_{b=1}^{100}\left({\hat{\beta }}_{j}^{b}-\beta_{j}\right)=\hat{\beta_{j}}-\beta_{j}$, where *b* refers to the subsample in the case-control data and the simulation study in the stimulation data. The mean squared error for a risk factor *x*_*j*_ is calculated as $\frac{1}{100}\sum_{b=1}^{100}{\left({\hat{\beta }}_{j}^{b}-\beta_{j}\right)}^{2}$.

#### Selection rate

The proportions of the selection for risk factors are also examined. For relevant variables, this corresponds to the true positive rate, and is thus positively oriented. For irrelevant variables, this corresponds to the false positive rate, and is thus negatively oriented.

R scripts for reproducing the methods and results for the simulation study are available in S3, S4 and S5 Files.

## Results

### Case-control data

**Fig 1:**
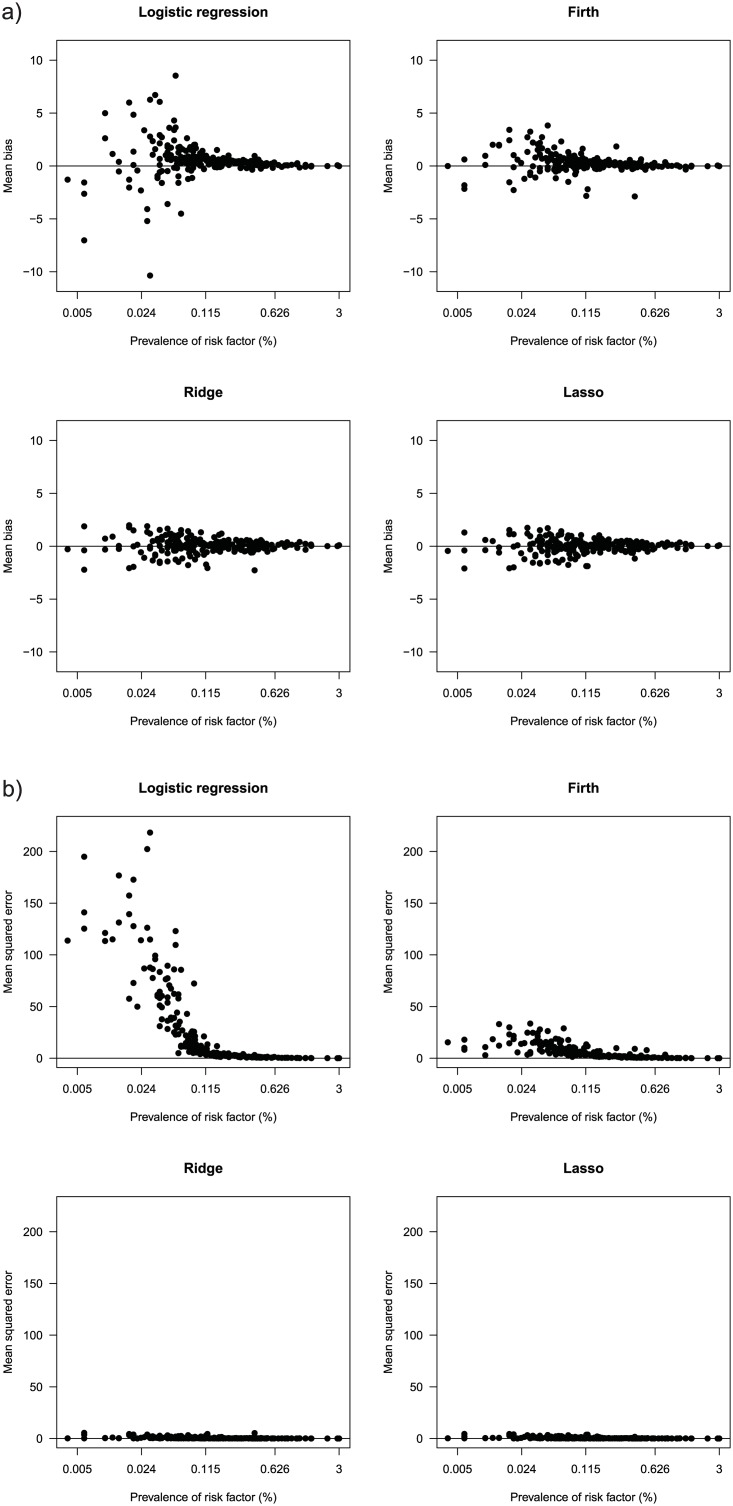
Case-control data. In the whole cohort of 50,728 drivers, we fit a reference model using logistic regression. We take 100 subsamples without replacement of 10,000 drivers and fit logistic, Firth, ridge and the lasso estimates. The bias (**1a**) and mean squared error (**1b**) of 213 risk factors shown are in comparison to the estimates of the reference model. The prevalence is on a log scale.

Fig 1a shows the mean biases of the 213 risk factors, sorted on the x-axis by prevalence. The standard logistic estimates are reasonably accurate down to a prevalence of about 0.1%, but risk factors with prevalences below 0.1% tend to be heavily biased. In contrast, Firth, ridge and the lasso have similar bias performances, with the distinct advantage that even rare risk factors tend to have plausible risk estimates. Even the 21 risk factors that were excluded due to their implausible estimates in the reference model all have plausible estimates if fitted with penalization which is also an argument in its favor.

Fig 1b compares the mean squared errors. Here, the difference between the standard logistic estimates and the penalized methods is even more extreme. Single exorbitant estimations from the standard logistic estimate inflate the mean squared error substantially. In contrast, the penalized methods show a much more consistent estimation and do not suffer from outliers amongst low exposure prevalences.

What is notable about these results is that the reference model is fitted using logistic regression, therefore one would expect that the standard logistic estimates on the subsamples has a large agreement with the reference model. However, the penalization methods have better agreement with the reference model because they incorporate a bias-variance trade-off, and therefore perform much better than standard logistic regression. In other words, penalization away from extreme estimates plays a larger role than correct model specification.

### Simulation data

Table 1 shows that among the relevant variables, the two most prevalent ones are selected most of the times by all methods. Starting with a prevalence of 0.1%, it is seen that the Firth correction and ridge regression do not select the variables as much as the other methods. The remaining methods perform similarly well, with the lasso having the best selection rate for extremely rare risk factors.

**Table 1:** CESIR simulation: Selection rates (%) for each of the 5 relevant variables.

	Prevalence of relevant variables				
	0.005%	0.02%	0.1%	0.6%	3%
**Logistic regression**	5	29	54	100	100
**Firth correction**	0	0	36	99	100
**Ridge regression**	0	4	37	97	100
**Lasso**	17	32	52	86	100
**Boosting**	0	8	60	100	100

Looking at the selection of irrelevant variables, Table 2 shows that the ridge regression performs best by not selecting the irrelevant variables most of the time. The lasso, which correctly selects the relevant variables, selects too many irrelevant variables. Logistic regression performs reasonably.

**Table 2:** CESIR simulation: Selection rates (%) for the 45 irrelevant variables, grouped together by prevalence (9 variables per interval).

	Prevalence of irrelevant variables				
	0.006%–0.016%	0.02%–0.06%	0.07%–0.22%	0.25%–0.8%	0.9%–3%
**Logistic regression**	10	21	18	15	15
**Firth correction**	0	1	7	11	11
**Ridge regression**	0	5	8	8	7
**Lasso**	24	35	36	33	36
**Boosting**	1	11	28	36	37

**Fig 2:**
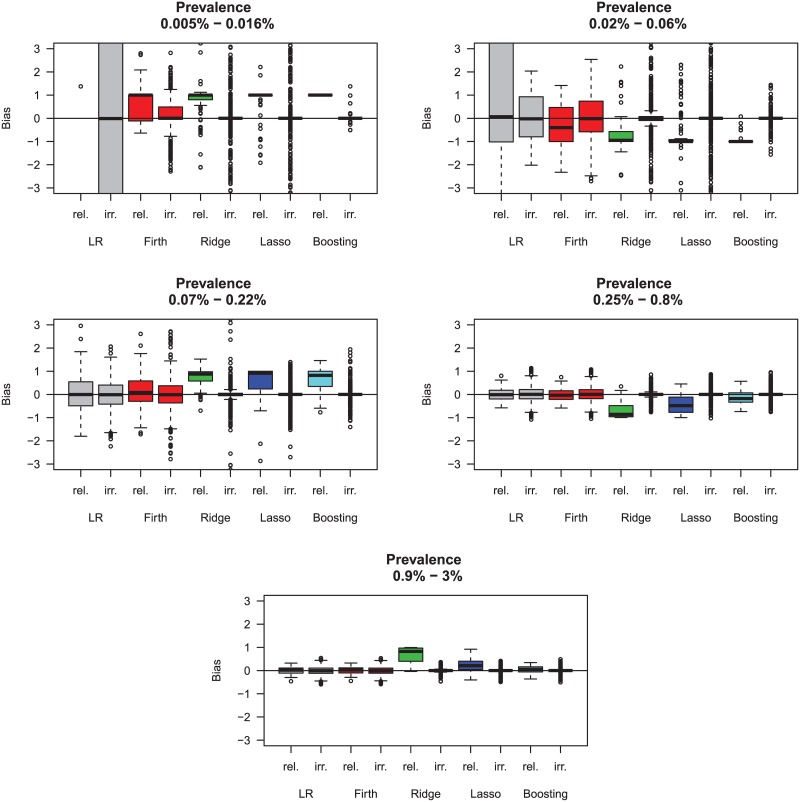
Simulation data. Each plot shows the bias for 1 relevant variable (rel.) and the 9 irrelevant variables (irr.) within the prevalence range. The boxplots are thus based on 100 values (one for each simulation study) for the relevant variables and 900 values (100*9) for the irrelevant variables.

Concerning the bias of the methods, Fig 2 shows that for higher prevalences from 0.9% to 3%, all methods have comparable performances, with some slight overestimation for relevant variables by ridge regression and the lasso. Starting from prevalences from 0.07% to 0.22%, the increase in bias is visible for all methods, with estimates tending to overestimate the true effect. Logistic regression and Firth correction perform best. For prevalences from 0.02% to 0.06%, the bias is starting to be substantial for most methods, or is exactly ±1 for the relevant variables (meaning that the relevant variables are not selected). Here, the Firth correction does very well, while boosting is strong in not selecting irrelevant variables. What is not always seen in these boxplots (in order to preserve the scale of the y-axis) are substantial outliers, particularly present for logistic regression.

**Fig 3:**
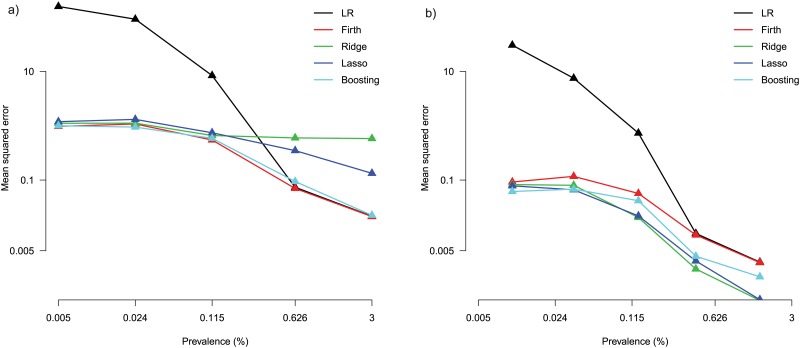
Simulation data. (a) Mean squared errors across the 5 relevant variables. (**b**) Mean squared errors across 5 groups of irrelevant variables (9 variables per group). All axes are on a logarithmic scale.

Fig 3a shows the problem of using logistic regression for rare variables—the variance of estimates is magnitudes greater compared to penalized methods. Boosting and Firth correction achieve the lowest mean squared errors. In Fig 3b, the variance of the estimates is lower compared to in Fig 3a. The penalization methods perform similarly well, with boosting being slightly better at extremely low prevalences. For both relevant and irrelevant variables, below a prevalence of 0.1% logistic regressions suffers from estimates that are larger by several orders of magnitude than those of the other estimation methods.

## Discussion

This work demonstrates the usefulness of penalized regression techniques for analyzing risk factors outside of high-dimensional settings. The focus was on the problem of risk factors with a very low prevalence, and it could be shown that standard techniques tend to fail as prevalence lowers. While a similar comparison was made for case-crossover studies by [15], here we explored these methods in the epidemiological setting of an unmatched case-control study.

For variable selection, the backward selection procedure based on standard maximum likelihood estimation was able to perform similarly well compared to the other methods. It has to be pointed out, however, that risk factors for which the likelihood of the standard logistic estimates did not converge were considered not selected. An alternative view could be that without convergence inference about the variable is not possible. For rare exposures with a prevalence less than 0.1%, logistic regression often failed to deliver meaningful log odds ratios estimates. Thus contrary to [9], bias did not become negligible with relatively large sample sizes for exposures that were extremely low. Firth and boosting showed the best behavior when dealing with (not extremely) low prevalence exposures, with Firth performing better at estimating relevant risk factors, and boosting being better at estimating the irrelevant risk factors. The impact of different effect sizes of relevant risk factors could be of interest as well, but in our work we decided to focus on prevalences, keeping effect sizes fixed.

When deciding which method to choose, other characteristics should be kept in mind which may be relevant to the problem, such as robustness to outliers, computational scalability, or predictive power, for which we refer to [12]. In our study, boosting was applicable to simulation data (of sample size *n* = 10,000) and failed to be applied to the real data (of sample size *n*>50,000). The key to all penalized regression methods is the degree of penalization, which is usually determined by the data; an exception here is the Firth correction, though a tuning approach may be worth investigating.

A strength of Firth regression is that standard errors are available for the estimates [4], making classical inference through p-values and confidence intervals possible. This is not the case for the lasso and boosting which lack natural expressions for their standard errors due to the adaptive nature of their estimation procedure. Recent suggestions have been made by [27] alternatively, this is sometimes compensated with resampling procedures. We show, however, that all penalized methods presented were useful because variable selection is provided through ways other than statistical significance.

It was noted that standard maximum likelihood methods produced considerable bias in our setting. Yet maximum likelihood methods are known to be consistent, meaning that under certain regularity conditions, the central limit theorem provides that maximum likelihood estimates converge to the true value of the parameter. It seems contradictory that penalized regression should thus improve estimation, because they introduce bias by definition. The distinction, however, is that consistency holds only for a sufficient amount of information (sufficiently large sample size, sufficiently balanced outcome and exposures), and therefore can be only of limited use in practice when sample size is finite and data is sparse. The strength of penalized methods compared to standard logistic regression can be explained by the bias-variance trade-off. Without penalization, the aim of the model is to minimize bias but the variance may be high. As penalization increases, the flexibility of the model decreases, leading to lower variance but higher bias. Through tuning, the penalization methods therefore seek to find a compromise between bias and variance, the effect of which is most notably seen when comparing mean squared errors which is a function of the variance and the squared bias.

Introducing a penalty term to the likelihood function can be thought of as a “budget constraint” that is placed on the risk factor estimates. Since penalized effect estimates tend to shrink, another term for penalized regression is “shrinkage”. A similar approach is to use Bayesian priors for the risk estimates and penalization is actually closely connected to priors [28]. While the penalty introduces a bias into the estimation process, we discussed earlier that estimation of rare risk factors is prone to gross overestimation, thus introducing a bias towards zero is a favorable counterbalance to this problem.

Numerous methods have been developed to assess all sorts of high-dimensional problems that may also be useful for low-dimensional settings, including ones with logistic regression for case-control studies [29]. While many newer techniques exist, mainly originating from machine learning, that are gaining popularity in biomedical research, many of these techniques are strong in predicting an outcome but the underlying relationship function remains a ‘black box’. The focus of this paper is on regression techniques because of their usefulness in providing a good interpretation of the influences of risk factors on the outcome, therefore increasing the understanding of the underlying relationship. Our results are relevant also for case-control studies with smaller *n*, as long as prevalences are low and high-dimensionality is not an issue (*n* > *p*).

## Conclusion

Our results showed drastic improvements in risk estimation for exposures with low and ultra-low prevalences, particularly using Firth and boosting. Because the problem of sparse risk factors is common, and because its implementation is available in most popular statistics softwares such as R, Stata, SAS and SPSS, we recommend using penalized regression when analyzing risk factors with low prevalences.

## Supporting information

S1 File Overview of the simulation study. (HTM)

S2 File Markdown file of the simulation study. (RMD)

S3 File R script to fit estimates to simulation study. (R)

S4 File R script to fit boosting estimates to simulation study. (R)

S5 File R script to produce results of the simulation study. (R)
