# Supplementary material for: Penalized logistic regression with low prevalence exposures beyond high dimensional settings
Source: PLoS One. 2019 May 20;14(5):e0217057. doi: 10.1371/journal.pone.0217057 (PMC6527211; doi:10.1371/journal.pone.0217057)
Supplement: S1 File — (HTM) [file pone.0217057.s001.htm]

CESIR - simulation study


# CESIR - simulation study

- 10,000 observations (5,000 cases, 5,000 controls)
  - CESIR: ~50,000 observations
  - chosen to be slightly smaller than CESIR to have fewer variables (e.g. when doing boxplots)
- 200 observations per variable => 50 variables
  - CESIR: 217 obs. per variable

```
n <- 10000
p <- n/200
```

- Prevalences will range from 3% (1 in 33.3) to 0.005% (1 in 20,000)
  - CESIR: similar prevalences

```
# We choose prevalences from 0.005% to 3% evenly on a log scale (which corresponds to the lognormal distribution in CESIR):
prevalence.sim <- exp(seq(from = log(0.03), to = log(0.00005), length.out = p))
prevalence.sim <- round(prevalence.sim, 5)
hist(x = prevalence.sim, breaks = 5, las = 1, xlab = "Prevalence", main = "Distribution of \n risk factor prevalences")
points(x = prevalence.sim[50:1], y = c(rep(36, 36), rep(5, 5), rep(3, 3), rep(2, 2), rep(2, 2), rep(2, 2)),
       pch = c(2, 17)[1 + 1:50 %in% c(1, 13, 26, 38, 50)], cex = c(1, 2)[1 + 1:50 %in% c(1, 13, 26, 38, 50)])
legend("topright", pch = c(17, 2), legend = c("relevant variable", "irrelevant variable"), bty = "n", pt.cex = c(2, 1))
```

- 10% of risk factors are relevant
  - In the CESIR data, about 10% of risk factors had a p-value less than 0.05 (in logistic model)

```
# From the 50 potential risk factors, We choose 5 relevant variables, evenly spaced from most frequent to most sparse
rel.index <- round(seq(from = 1, to = p, length.out = p*0.10))
rel.index
```

```
## [1]  1 13 26 38 50
```

- low correlation of risk factors
  - In the CESIR data, there were only few correlated drugs, therefore the assumption is valid

```
# The matrix of common probabilities:
mat <- outer(X = prevalence.sim, Y = prevalence.sim)
mat[lower.tri(mat)] <- NA # to make matrix symmetric
# set.seed(1) # the seed should be set in run_simulation.R
mat <- mat * 2 ^ rnorm(n = length(mat), mean = 0, sd = 0.25)
mat[lower.tri(mat)] <- t(mat)[lower.tri(mat)] # to make matrix symmetric
diag(mat) <- prevalence.sim

library(bindata)
# We first generate a large population (i.e. the whole country's population) of people in a road traffic accident
n.large <- n*2
X.large <- rmvbin(n = n.large, commonprob = mat)
```

- Baseline risk (intercept) for the population will be 0
  - this was also observed in the CESIR database (Avalos et al. Epidem 2012)
  - of all the drivers in a road traffic accident with another person, half of them will be responsible
  - if accident happens alone, it’s also fair to assume that half the time the driver is responsible
- The risk factors will have effect size 1
  - CESIR: most effect sizes range from -1 to 1
- of the 5 relevant risk factors, 3 will be negative and 2 will be positive
  - CESIR: of the 23 significant risk factors, 17 have negative coefficient and 6 have positive coefficient
- unobserved confounder z with prevalence 1%
  - Assumption by Avalos et al. (Stat Med 2012)
  - effect size chosen so that signal to noise ratio of 3 (Avalos et al., Stat Med 2012)

```
beta <- c(-1, 1, -1, 1, -1)
Var.Xb <- sum(apply(X.large[, rel.index], 2, var) * beta ^ 2)

q <- 2
library(bindata)
z.large <- matrix(rbinom(n = n.large*q, size = 1, prob = 0.5), nrow = n.large, ncol = q)
# s.n.ratio <- Var.Xb/Var.Z
s.n.ratio <- 3
gamma <- sqrt(Var.Xb/sum(apply(z.large, 2, var) * s.n.ratio))
gamma <- rep(x = gamma * c(1, -1), length.out = q)
Var.Z <- sum(apply(z.large, 2, var) * gamma ^ 2)

eta <- X.large[, rel.index] %*% beta + z.large %*% gamma
y.large <- rbinom(n = n.large, size = 1, prob = binomial()$linkinv(eta))

table(y.large)
```

```
## y.large
##     0     1 
## 10069  9931
```

```
# From the large population, we now create an index of the first 5000 cases (index.1) and the first 5000 controls (index.0):

index.1 <- which(y.large == 1)[1:(n/2)]
index.0 <- which(y.large == 0)[1:(n/2)]

X1 <- X.large[index.1, ]
X0 <- X.large[index.0, ]

X <- rbind(X1, X0)
dim(X)
```

```
## [1] 10000    50
```

```
z <- c(z.large[index.1, ], z.large[index.0, ])
      
y <- c(rep(x = 1, times = n/2), rep(x = 0, times = n/2))

table(y)
```

```
## y
##    0    1 
## 5000 5000
```

```
rm(X.large, X0, X1, index.0, index.1, n.large, y.large, z.large)
```
